# Brazilian generics market change after Farmácia Popular program

**DOI:** 10.11606/s1518-8787.2019053001237

**Published:** 2019-10-10

**Authors:** Andréa Dâmaso Bertoldi, Luisa Arueira Chaves, Dennis Ross-Degnan, Vera Lucia Luiza, Isabel Cristina Martins Emmerick, Rondineli Mendes da Silva, Mônica Rodrigues Campos

**Affiliations:** I Universidade Federal de Pelotas . Faculdade de Medicina . Programa de Pós-graduação em Epidemiologia . Pelotas , RS , Brasil; II Universidade Federal do Rio de Janeiro , Campus Macaé . Macaé , RJ , Brasil; III Harvard Medical School & Harvard Pilgrim Health Care Institute . Department of Population Medicine , Drug Policy Research Group. Boston , MA , USA; IV Fundação Oswaldo Cruz . Escola Nacional de Saúde Pública Sergio Arouca . Departamento de Política de Medicamentos e Assistência Farmacêutica . Rio de Janeiro , RJ , Brasil; V University of Massachusetts Medical School . Department of Surgery . Division of Thoracic Surgery . Worcester , MA , USA; VI Fundação Oswaldo Cruz . Escola Nacional de Saúde Pública Sergio Arouca . Departamento de Ciências Sociais . Rio de Janeiro , RJ , Brasil

**Keywords:** Drugs, Generic, economics, Generic Drug Policy, Antihypertensive Agents, supply & distribution, Hypoglycemic Agents, supply & distribution

## Abstract

**OBJECTIVE:**

To evaluate trends in the use of generic and non-generic medicines to treat hypertension and diabetes under the *Farmácia Popular* Program (FP) and its impact on generic medicines sales volume and market share in the Brazilian pharmaceutical market.

**METHODS:**

This longitudinal, retrospective study used interrupted time series design to analyze changes in monthly sales volume and proportion of medicines sales (market share) for oral antidiabetic and antihypertensive medicines for generic *versus* non-generic products. Analyses were conducted in a combined dataset that aggregate monthly sales volumes from the *Farmácia Popular* program and from the QuintilesIMS™ (IQVIA) national market sales data from January 2007 to December 2012. The *Farmácia Popular* program phases analyzed included: a) 2009 reductions in medicines reference prices (AFP-II) and b) 2011 implementation of free medicines program for hypertension and diabetes, the *Saúde não tem preço* (SNTP – Health has no price).

**RESULTS:**

Patterns of use for FP-covered antidiabetic and antihypertensive medicines were similar to their use in the market in general. After one year of the decreases in government subsidies in April 2010, market share of antidiabetic and antihypertensive medicines experienced relative declines of -54.5% and -59.9%, respectively. However, when FP-covered medicines were made free to patients, overall market volume for antidiabetic and antihypertensive generics increased dramatically, with 242.6% and 277.0% relative increases by February 2012, as well as non-generics with relative increase of 209.7% and 279% for antidiabetic and antihypertensive medicines, respectively.

**CONCLUSIONS:**

Ministry of Health policies on the amount of patient cost sharing and on the choice of medicines on coverage lists have substantial impacts on overall generic sales volume in retail pharmacies.

## INTRODUCTION

Access to medicines is one of the key elements in ensuring the right to health. However, its high burden in health systems budgets, especially in low and middle-income countries, represents a challenge to be overcome ^1^ . One of the strategies meant to reduce the cost of medicines is the use of generics. Generics are defined as “(…) *a pharmaceutical product, usually intended to be interchangeable with an innovator product* (…)” ^a^ .The World Health Organization (WHO) recommends facilitating early market entry of generics and allowing substitution by dispensers to reduce medicine budgets and improve access to medicines ^2^ .

Brazil is a country that recognizes the right to health, including access to medicines as a right of its citizens ^3^ . In 1998, the Brazilian National Medicines Policy promoted the use of generics by several strategies ^4^ . In 1999, Brazil published a generics law. Unless explicitly stated in a prescription, all medicines can be substituted by their generic version ^5^ . Since then, the use of generics has been growing and a recent national household survey demonstrated that about half of the interviewees (45.5%) used at least one generic medicine in treating chronic diseases or a recent acute illness ^6^ .

Brazilian public health facilities dispense medicines found in the *Relação Nacional de Medicamentos Essenciais* (Brazilian Essential Medicines List) free of charge at dispensing. Until 2004, that was the only governmental system for patients to receive these medicines. However, about 29% of the Brazilian population are covered by private health insurance plans ^7^ that, in general, do not cover medicines in their benefits. To reduce the financial burden of these families related to the purchasing of medicines, the Brazilian federal government launched the *Farmácia Popular* program (FP) in 2004, in which a reference list of medicines are provided to all citizens through a subsidy system that the patient is responsible for paying a share of the medicines’ cost ^8^ . Different from the previous public mechanism, the FP accepts prescriptions from any authorized public or private prescriber. The medicines were provided in specific public establishments distributed across the country ^9^ .

In 2006, a subset of the medicines provided in public FP establishments were also made available in private retail pharmacies ^10^ . This change has resulted in a rapid geographic expansion of availability, although outlets remain unequally distributed across Brazilian regions ^9^ . FP underwent other important policy changes in 2009, 2011 and 2012. In 2009, there were some important administrative changes ^11^ . In 2011, to improve the treatment of non-communicable diseases, medicines for hypertension and diabetes began to be offered free of charge to patients (100% subsidy) in both public and private FP outlets. This strategy was called *Saúde não tem preço* (SNTP – Health has no price ) ^12 , 13^ . In all phases of the FP, one objective was to promote use of generics, in agreement with the National Medicines Policy.

This article was developed under a broader study called “Impact of consecutive subsidies policies on access to and use of medicines in Brazil (ISAUM-Br Project)” ^14^ . The main project goal was to evaluate the impact of the Brazilian FP subsidy policy on access and use of medicines. Our aim in this article is to evaluate trends in use of generic and non-generic medicines to treat hypertension and diabetes under the FP and its impact on generic medicines sales volume and market share in the Brazilian pharmaceutical market.

## METHODS

This study focuses on three phases of the FP. From 2006 to 2009 (phase I *Aqui tem Farmácia Popular* I, – AFP-I – *Farmácia Popular* is available here), FP medicines were made available in private pharmacies with low levels of patient copayments. In April 2009 (phase AFP-II), the government reduced reference prices for most FP medicines by 24.5% on average. This change resulted in an immediate increase in patient copayment from about 2.45 to 3.88 *reais* per 30 days dispensing, a relative 58.4% increase. The second change in February 2011 (SNTP) involved government implementation of fully subsidized coverage for medicines for hypertension and diabetes, which were offered with no copayment from patients.

This article based its analysis on two different datasets: the FP reimbursement data and QuintilesIMS™ (IQVIA) national market sales data. FP data were obtained from an electronic point-of-sales dispensing program in retail pharmacies. Data include information about the patient and the medicines such as age and gender, geographic location of pharmacies, medicine dispensed and date of dispensing, including classification as generic, originator, or *similares* ; daily-prescribed dose; Ministry of Health reimbursement and patient copayment amount. This article used data on sales volume for hypertension and diabetes medicines from January 2007 to December 2012. Dispensing data are of good quality and relatively complete.

The IQVIA data ^14^ available to this project comprise the monthly sales volume and prices for all oral hypertension and diabetes medicines from 2002 to 2013 at country level. Available information includes: Therapeutic Class Levels 2 and 4 (European Pharmaceutical Market Research Association (EphMRA) Anatomical Classification of Pharmaceuticals ^15^ ); manufacturer; molecule; whether generic, reference-originator, *marca* or *similar* (generic classification under Brazilian law); presentation; years of market entry of the product and presentation. The sales data were clean and contained no relevant missing information.

The generic classification was standardized in both data sets for analysis in this study. Products were classified as generic or non-generic, considering that the latter includes originators and *similares* in the FP data and originators, *marca* and *similares* in the IQVIA data.

In Brazilian regulation, generics are defined as medicines that meet clinical equivalence criteria, are commercialized under the international nonproprietary name (INN) and are considered interchangeable with the originator brand product. Originators are the first brand-name product registered, whereas *similares* or *marca* are non-originator brand-name products equivalent to the originator, however, their bioequivalence has not been proven ^16^ . Bioequivalence has to be proved since 2014 ^17^ .

In the IQVIA dataset, medicines were classified as covered by the FP or non-covered, the latter of which is used as a control group. The sales of single ingredient formulations accounted for more than 85% of the sales volume of diabetes and hypertension medications, and for that reason combination products were excluded from this analysis.

The primary outcome variables were monthly sales volumes for generic and non-generic products (measured as number of pills) for both oral antidiabetic medicines and antihypertensive medicines. The four oral antidiabetic medicines covered by FP are glibenclamide 5 mg, and metformin 500mg, 850mg, and slow release 500mg; and the six antihypertensive medicines are atenolol 25 mg, propranolol 40 mg, hydrochlorothiazide 25 mg, captopril 25 mg, enalapril 5 mg, and losartan 50 mg. Market volumes for both generic and non-generic products were summarized in both data sources; in the IQVIA data, the volumes for products covered and non-covered were also summarized.

Using these primary outcome variables, five measures of generic market share and AFP market share were created, as follows:

- FP volume as a proportion of market volume for generics (only FP covered medicines): volume of generics covered by FP divided by the total market volume of generics in IQVIA;
- FP volume as a proportion of market volume for non-generics (only FP covered medicines): volume of non-generics covered by FP divided by the total market volume of non-generics in IQVIA;
- FP generic volume as a proportion of total FP volume (all covered medicines): volume of generic medicines covered by FP divided by the total volume of medicines covered in FP;
- Generic volume as a proportion of market volume (FP covered medicines): volume of covered generic medicines in IQVIA divided by the total volume of all medicines in IQVIA;
- Generic volume as a proportion of market volume (FP non-covered medicines): volume of generic medicines in IQVIA not covered by FP divided by the total volume of all medicines in IQVIA.

This study applied interrupted time series (ITS) segmented linear regression models in the analyses to determine the effect of the FP policy changes on our study outcomes. ITS models adjust for pre-existing trends in the period before each policy change. The *prais* command in Stata v12 was used.

The ITS models included three segments – one per program period – with 27, 20, and 21 monthly observations. It was estimated that it would take two months for the full effects of each policy to be reflected on the volumes sold, so the two immediate post-policy months were set as missing in all analyses. Each policy effect was estimated by one variable representing the change in level of the outcome immediately after the policy and a second representing the change in trend of the post-policy segment.

All parameters were retained in the models regardless of statistical significance. Statistically significant results (p < 0.05) were highlighted. To create single number summaries of policy effects, estimates of the relative changes in outcomes compared to expected values were calculated based on prior trends for April 2010 and February 2012, about one year after each of the policy interventions.

## RESULTS

During the entire study period, FP accounted for a substantial share of the overall Brazilian market for oral antidiabetic and antihypertensive medicines ( Table 1 , a and b). For the oral antidiabetic, FP represented over two-fifths of the generics market share (43.9%) and one-third of the non-generics (35.5%) prior to AFP-II. The scenario for antihypertensive was different since sales within FP both for generics (36.3%) and non-generics (32.3%) were about one-third of the total market share prior to the AFP-II intervention. When AFP-II started, the reference prices of medicines were reduced, and part of the cost was shifted to patients. FP market share for oral antidiabetic medicines decreased substantially for generics (34.3%), while the non-generic market share stayed quite stable (33.5%). Antihypertensive drugs also experienced decreases in FP market share, but for this class of medicines, both versions – generics and non-generics – had similar reductions (25.5% and 26.6%, respectively). The SNTP dramatically affected the FP generics market share. Oral antidiabetic generics in FP doubled their market share, reaching 69.8% of the total market of the covered medicines; the same was observed for antihypertensive generic medicines, which reached 61.9% of total market share after this intervention. The non-generic versions of oral antidiabetic and antihypertensive medicines within the FP also had their market share increased, although not in the same magnitude as observed for generics, reaching 56.9% and 37.5%, respectively.

**Table 1:** Average proportions of medicines volume for generic, non-generic, covered and not covered, by stage of the private sector *Farmácia Popular* program, Brazil, 2007 to 2012.

List of indicators	Oral antidiabetic medicines (%)			Antihypertensive medicines (%)		
	
	AFP-I	AFP-II	SNTP	AFP-I	AFP-II	SNP
					
	(Jan 2007–Mar 2009)	(Apr 2009–Jan 2011)	(Feb 2011–Dec 2012)	(Jan 2007–Mar 2009)	(Apr 2009–Jan 2011)	(Feb 2011–Dec 2012)
a. AFP volume as a proportion of market volume: generics (only AFP covered medicines)	43.9	34.3	69.8	36.3	25.5	61.9
b. AFP volume as a proportion of market volume: non-generics (only AFP covered medicines)	35.5	33.5	56.9	32.3	26.6	37.5
c. AFP generic volume as a proportion of total AFP volume* (all covered medicines)	70.1	58.8	61.6	56.3	53.1	77.3
d. Generic volume as a proportion of market volume (AFP covered medicines)	63.6	58.2	56.7	49.9	53.8	67.6
e. Generic volume as a proportion of market volume (AFP non-covered medicines)	20.2	26.1	30.6	24.1	27.1	33.7

Indicators calculation formula:

1. volume of generics covered in AFP divided by the total market volume of generics in IQVIA

2. volume of non-generics covered in AFP divided by the total market volume of non-generics in IQVIA

3. volume of generic medicines covered in AFP divided by the total volume of medicines covered in AFP

4. volume of covered generic medicines in IQVIA divided by the total volume of all medicines in IQVIA

5. volume of generic medicines in IQVIA not covered by AFP divided by the total volume of all medicines in IQVIA

AFP: “ *Aqui tem Farmácia Popular* ” private sector program, phases I and II; AFP-I expansion for private sector (2006), here analyzed from January 2007 to March 2009; AFP-II – reduction of reference prices and consequent increase in patient copayment from April 2009 to January 2011; SNTP: “ *Saúde não tem preço* ” (Health has no price) phase. All covered medicines for hypertension and diabetes being free of charge to patients starting on February 2011; IQVIA = QuintilesIMS™

* Total AFP volume = Generic + Non-generic sales volume in pharmaceutical units.

Most dispensing within the FP has been generic medicines, reaching 70.1% of oral antidiabetic and 56.3% for antihypertensive during AFP-I. After AFP-II, there was a decline in generics sales within the program, especially for oral antidiabetic ones, which had their share reduced to 58.8% at this time. In contrast, antihypertensive medicines experienced only a small reduction, representing 53.1% of the sales during this period. With SNTP, oral antidiabetic and antihypertensive generics again increased as a proportion of sales, representing 61.6% and 77.3% of FP sales, respectively ( Table 1 , c).

Generic market share for medicines covered by FP in the overall Brazilian market, based on IQVIA data ( Table 1 , d), mirrors the generic market share in FP. During AFP-I, oral antidiabetic and antihypertensive generics represented 63.6% and 49.9%, respectively, of the sales in the Brazilian market. After AFP-II, while generics for oral antidiabetic experienced about a 5% reduction (58.2%), antihypertensive generics market share increased by a similar amount (53.8%). The SNTP did not appear to change overall oral antidiabetic generic market shares appreciably (56.7%), but antihypertensive generic market share increased substantially (67.6%). In contrast to the patterns of generic market share for medicines covered by the AFP, generic market share among medicines that are not covered by the program ( Table 1 , e) was much lower during the study period, representing only 20.2% and 24.1% during AFP-I, 26.1% and 27.1% during AFP-II, and 30.6% and 33.7% during SNTP, for oral antidiabetic and antihypertensive, respectively.

**Figure 1:**
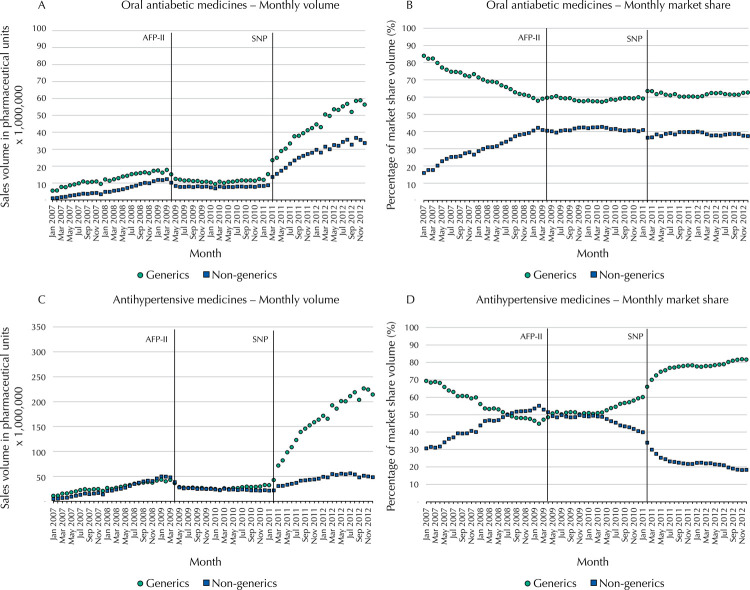
Monthly volume and market share of oral antidiabetic and antihypertensive medicines in the *Farmácia Popular* program, by generic and non-generic (AFP dataset), Brazil 2007 to 2012.

AFP: *Aqui tem Farmácia Popular* private sector program, phases I and II; AFP-II – reduction of reference prices and consequent increase in patient copayment from April 2009 to January 2011; SNTP: *Saúde não tem preço* (Health has no price) phase. All covered medicines for hypertension and diabetes being free of charge to patients starting from February 2011; IQVIA: QuintilesIMS™; MS: Market share

Figure 1 compares the sales volume and market share of generics and non-generics within the FP database from 2007 and 2012. Regarding sales volume ( Figure 1 , A and C), both generics and non-generics had increasing sales volume prior to the AFP-II policy in 2009, after which both experienced small reductions in sales volume. However, after free medicines became available following the SNTP policy, generics and non-generics both had impressive increases in sales volume, with a greater magnitude of increase for generics. Oral antidiabetic and antihypertensive had similar trends over time. However, generics always had higher sales volume for oral antidiabetic medicines, while for antihypertensive ones, generic sales volume only surpassed non-generic sales following SNTP, when antihypertensive generics experienced a greater relative increase in volume than oral antidiabetic medicines.

At the beginning of the study period, generics dominated market share for both antihypertensive and oral antidiabetic medicines ( Figure 1 , B and D), although their share declined prior to the AFP-II policy. Oral antidiabetic generics represented 85% of the market in 2006, which declined to 60% by 2009, where it stabilized throughout the period of the AFP-II and SNTP interventions. For antihypertensive medicines ( Figure 1 , D) just before AFP-II, non-generics overtook generics to constitute a majority of the market; after AFP-II, generic market share stabilized at around 50% but began to increase again in mid-2010 to reach approximately, 60% of the market. After SNTP, antihypertensive generic market shares increased again, eventually stabilizing at about 80% of the market.

**Figura 2:**
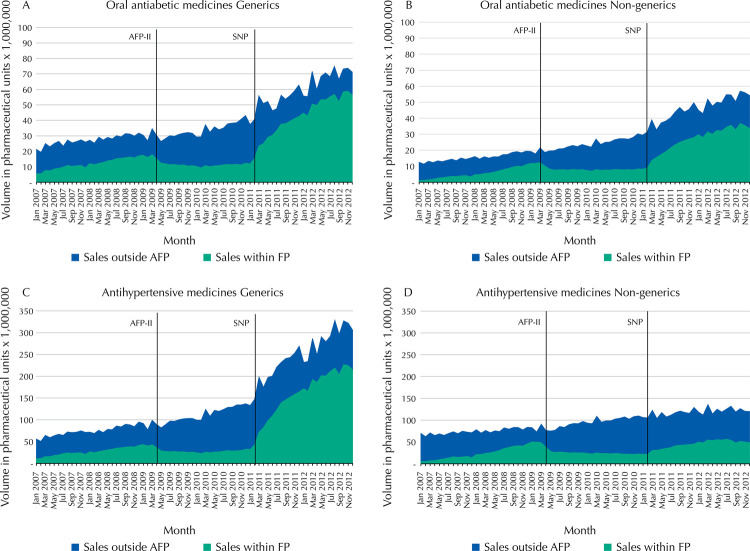
Monthly volume of oral antidiabetic and antihypertensive medicines dispensed in the AFP program in comparison to volumes in the total Brazilian market, by generic and non-generic medicines covered by the program (AFP and IQVIA dataset*), 2007 to 2012.

**Figure 3:**
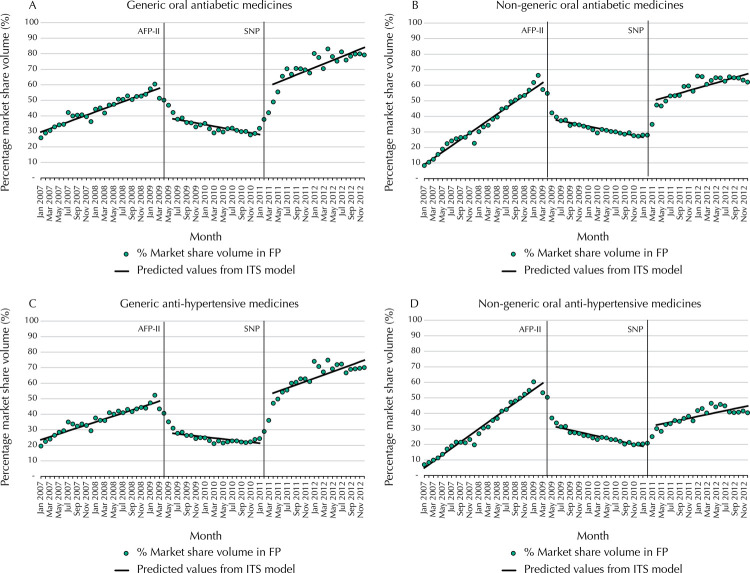
Monthly market share* for generic and non-generic oral antidiabetic and antihypertensive medicines in the overall Brazilian market and predicted values from segmented regression models (AFP and IQVIA dataset), 2007 to 2012.

AFP: *Aqui tem Farmácia Popular* private sector program, phases I and II; AFP-II – reduction of reference prices and consequent increase in patient copayment from April 2009 to January 2011; SNTP: *Saúde não tem preço* (Health has no price) phase. All covered medicines for hypertension and diabetes being free of charge to patients starting from February 2011; IQVIA: QuintilesIMS™; MS: Market share; A, B, C, and D: indicators presented in Table 2.

Figure 2 shows the volume of FP sales for covered oral antidiabetic and antihypertensive medicines within the total Brazilian market, for both generics and non-generics. Figure 3 and Table 2 present time series estimates of the changes in FP market share as a percentage of the total sales volume for these medicines in the three study phases.

*For this graph, AFP dataset indicates the Sales within AFP and IQVIA dataset indicates all sales in the Brazilian market of AFP covered medicines.

AFP: *Aqui tem Farmácia Popular* private sector program, phases I and II; AFP-II – reduction of reference prices and consequent increase in patient copayment from April 2009 to January 2011; SNTP: *Saúde não tem preço* (Health has no price) phase. All covered medicines for hypertension and diabetes being free of charge to patients starting from February 2011; IQVIA: QuintilesIMS™; MS: Market share

* Market share (MS) is calculated as the volume of medicines sold in AFP divided by the total market volume sold in IQVIA data, for oral antidiabetic and antihypertensive generic and non-generic medicines that are covered in the AFP program

**Table 2:** Monthly market share for oral antidiabetic and antihypertensive medicines, generic and non-generic, and changes in market share level and trend by stage of the *Farmácia Popular* program, Brazil, 2007 to 2012.

Market share measure ^a^	Baseline		AFP II (April 2009)				SNTP (February 2011)			
		
	MS (Jan 2007)	Trend	MS (Mar 2009)	Level change after policy (95%CI)	Trend change after policy (95%CI)	% relative change in MS (Apr 2010)	MS (Jan 2011)	Level change after policy (95%CI)	Trend change after policy (95%CI)	% relative change in MS (Feb 2012)
Diabetes										
A) AFP volume as a proportion of market volume: generics ^b^	28.5	1.1	57.8	-21.3 (-27.6 – -15.0)	-1.6 (-2.1 – -1.2)	-54.4	28.0	32.3 (25.5 – 39.0)	1.7 (1.2 – 2.2)	242.6
B) AFP volume as a proportion of market volume: non-generics ^b^	6.9	2.0	61.7	-27.5 (-32.9 – -22.1)	-2.6 (-3.0 – -2.2)	-63.9	26.6	24.4 (18.6 – 30.2)	1.4 (1.0 – 1.9)	209.7
Hypertension										
C) AFP volume as a proportion of market volume: generics ^b^	22.6	1.0	48.4	-22.3 (-28.8 – -15.8)	-1.3 (-1.8 – -0.8)	-59.9	21.4	31.8 (24.9 – 38.7)	1.4 (0.9 – 1.9)	277.0
D) AFP volume as a proportion of market volume: non-generics ^b^	2.8	2.1	59.5	-31.7 (-37.4 – -26.1)	-2.8 (-3.2 – -2.4)	-71.5	18.8	14.4 (8.4 – 20.5)	1.3 (0.8 – 1.7)	279.0

AFP: *Aqui tem Farmácia Popular* private sector program, phases I and II; AFP-II – reduction of reference prices and consequent increase in patient copayment from April 2009 to January 2011; SNTP: *Saúde não tem preço* (Health has no price) phase. All covered medicines for hypertension and diabetes being free of charge to patients starting from February 2011; IQVIA: QuintilesIMS™; MS: Market share

^a^ Market share (MS) is calculated as the volume of medicines sold in AFP divided by the total market volume sold in IQVIA data, for oral antidiabetic and antihypertensive generic and non-generic medicines that are covered in the AFP program; They represent the indicators a and b presented in the Table 1.

^b^ A, B, C, and D correspond to the graphs in Figure 3.

Values in bold present p < 0.05

For oral antidiabetic and antihypertensive generics ( Figures 2 and 3 , Table 2 , sections A and C), FP accounted for an increasing proportion of the market, reaching about half of total sales for covered medicines prior to AFP-II. After this policy change, the overall market continued to grow slightly, but the proportion of sales within FP decreased in both level and trend, representing relative declines in market share of -54.5% and -59.9% for oral antidiabetic and antihypertensive medicines, respectively, by April 2010. However, after the SNTP policy, overall market volume for oral antidiabetic and antihypertensive generics increased dramatically, as did FP market share, with relative increases of 242.6% and 277.0% by February 2012. By the end of the study period, FP generics represented the vast majority of market sales for covered medicines in these two classes.

Non-generic oral antidiabetic ( Figures 2 and 3 and Table 2 , section B) had similar patterns of effects as generics, with effects differing mainly in magnitude. By the end of the study period, the FP market share was slightly less than 70%. In contrast, non-generic antihypertensive medicines ( Figures 2 and 3 and Table 2 , section D) continued to have a greater proportion of sales outside of FP than inside the program. In addition, the SNTP policy was not associated with large changes in market size.

## DISCUSSION

Within the FP, generics have represented the largest share of its sales since the beginning, suffering a decline during AFP-II when patient copayments increased, but increasing markedly after covered medicines became free to patients following SNTP. In the end of 2012, generics represented about 60% of all oral antidiabetic medicines and more than three-fourths of antihypertensive sales in the program. The AFP program accounts for an important and increasing share of the overall antihypertensive and oral antidiabetic market. This program represented about one-third of the market for both generics and non-generics prior to AFP-II, although market share declined somewhat during AFP-II when patient cost sharing was higher. FP utilization increased dramatically during the SNTP phase of the program, such that FP dispensing had a 35.5% and 36.4% growth for the generic market, and 23.4% and 10.9% growth for the non-generic market for oral antidiabetic and antihypertensive medicines, respectively. That means that FP also increased the volume of use of covered non-generic medicines.

Zero copayment lead many more patients to fill through the FP program, as well as to large increases in generics market share either both inside the program and in the overall market, implying either that there are more people under treatment, better adherence, or both. In fact, results from the ISAUM-Br Project ^18^ have shown that both phenomena occurred: more people started to participate in the program and the proportion of days covered per patient (PDC, a proxy measure for adherence) increased as well.

Policies that promote the use of generics offer a way to increase the efficiency of expenditures on medicines by encouraging the use of quality-assured medicines at lower costs ^19^ . In the Brazilian context, prescriptions at the public health system must be written using INN, a policy with reasonable adherence by prescribers ^20^ . It is also recommended that private medics prescribe using INN, although not mandatory. Moreover, even if the prescription does not follow INN, substitution by the generic version can be done at dispensing by the pharmacist ^23^ . Generic medicines’ prices must be 35% lower than the originator in the national market, and since the prices of medicines in Brazil are regulated, it must be declared at registration ^24^ .

All antihypertensive and oral antidiabetic medicines in FP have generic versions in the Brazilian market. A recent Brazilian survey on access and utilization of medicines showed that about 40% of all oral antidiabetic and antihypertensive medicines in Brazil are generics ^6^ . Comparing this finding with our results, generic utilization in FP is greater than the national average. Generics, especially after SNTP, have been prioritized in the program.

In this study, we have analyzed generics in comparison with non-generics, which includes both originator and branded generics. In Brazil, branded generics do not have to comply with the same price regulations as generics; however, since 2014 branded generics have been required to prove their bioequivalence in relation to the originator.

Since FP financing is based on a reference price established by INN and generic substitution can be done at dispensing, one can hypothesize that private pharmacies participating in the program might prefer to dispense generics to increase revenues due to their lower costs in the market. However, this hypothesis may not be true; one study done in 2009 showed that branded generics can have lower prices than generics in Brazil ^25^ . If pharmacies choose the lowest cost version to increase revenue, the choice may be branded generics and not generics. This may be an indirect consequence of generics policy enforcement.

The administrative changes implemented in AFP-II, which included a reduction in the reference price, had a negative impact on FP sales volume for all medicines analyzed. This reduction represented a shift of cost to patients, at that time, for 10% of the reference price of a medicine plus any differences between the reference price and the actual medicine sales price. This policy might have stemmed from increased audit mechanisms implemented during this period after denouncement of fraud in FP sales ^26^ . These mechanisms included linkage between different information systems and a maximum limit of medicines per patient based on clinical treatment protocols ^9 , 27^ .

The implementation of the SNTP was accompanied by a sharp increase in sales volume for all medicines. After SNTP, generics market share increased impressively for all medicines studied, suggesting that generics have been the drugs of choice for retail pharmacies.

Although similar, the results for oral antidiabetic and antihypertensive medicines have interesting differences; most antihypertensive medicines are sold outside FP, which differs from what is observed for oral antidiabetic ones. The most sold antihypertensive in Brazil in 2010 and 2011 (valsartan) is not on the FP list, so it can only be bought outside the program. Furthermore, the first generic version of this medicine was only available in the Brazilian market in February 2011 ^28 , 29^ . For oral antidiabetic medicines, the most sold active ingredients have always been present in the FP list.

Given the Brazilian definition of generic medicine used in our study, the international comparison of our findings is limited, since branded generics were considered non-generics.

These results demonstrate the complex relationships that access to medicines policies have with other components of the health system, as suggested by the access to medicines framework proposed by Bigdeli et al. ^30^ The FP program is a good model for these complex interactions since the program has used an innovative approach for health financing, developed and implemented a new information system, affected market forces and service delivery, and demanded a governance structure to manage and monitor the program.

Relevant future studies could address if the impact of FP on other medicines groups; the users’ profile, including source of health care; health outcomes; and cost effectiveness between FP and free-of-charge provision in SUS.

The *Farmácia Popular* program has had a great impact on the overall generics market, as well as on the expansion of the pharmaceutical market in Brazil through private retail pharmacies. Thus, government coverage policies for important classes of medicines can be used as a powerful tool to drive the overall market and increase generic market share.

## Footnotes

a World Health Organization. Measuring medicine prices, availability, affordability and price components. Geneva: WHO; 2008 [cited 2019 Jul 11]. Available from: https://www.who.int/medicines/areas/access/OMS_Medicine_prices.pdf
